# Risk Factors Associated with Loss to Follow-Up during Tuberculosis Treatment in the Sanatorium Hospital of Luanda, Angola

**DOI:** 10.3390/tropicalmed9060131

**Published:** 2024-06-12

**Authors:** Domingos Vita, Maria Luisa Aznar, Joan Martínez-Campreciós, Debora Cristina Maindo Sebastiao Kansietoko, Israel Molina

**Affiliations:** 1 DSS/EMG/FAA-Angola, Vita International Health Agency, London SW8 4EP, UK; 2 Department of Engineering and Technology, Instituto Superior Politécnico de Tecnologias e Ciências (ISPTEC), Luanda 2850, Angola; 3 Instituto Superior Técnico Militar (ISTM), Luanda 2850, Angola; 4 Department of Forensic Science, Geeta University, Panipat 132145, India; 5 International Health Unit Vall d’Hebron-Drassanes, Infectious Diseases Department Vall d’Hebron University Hospital, PROSICS, 119-129, 08034 Barcelona, Spain; marialuisa.aznar@vallhebron.cat (M.L.A.); joan.martinez@vhir.org (J.M.-C.); Israel.molina@vallhebron.cat (I.M.); 6 Centro de Investigación Biomédica en Red de Enfermedades Infecciosas (CIBERINFEC), Instituto de Salud Carlos III, 28029 Madrid, Spain; 7 Complexo Hospitalar Cardio Pulmonar, Cardeal Dom Alexandre de Nascimento, Luanda 2850, Angola; dekansietoko@gmail.com

**Keywords:** tuberculosis, loss to follow-up, food insecurity

## Abstract

Background: Tuberculosis (TB) continues to be a serious public health threat that affects the most vulnerable populations. Patients who are lost to follow-up (LTFU) after TB diagnosis still represent one of the biggest challenges to TB control. Method: In this prospective observational study, we aimed to identify and analyse the risk factors associated with LTFU among TB patients who started first-line TB treatment in the Sanatorium Hospital in Luanda. Result: A total of 113 patients with TB (non-multidrug resistant) were included between August 2018 and September 2019. Seventy-six (67.3%) patients were cured, 27 (23.9%) were LTFU, 5 (4.4%) died, 4 (3.5%) were transferred and 1 (0.9%) presented treatment failure. After excluding those who died, were transferred or failed treatment, we observed that severe TB at the time of diagnosis (OR 9.24, 95% CI 2.18–39.04) and food insecurity were significantly associated with LTFU (OR 5.96, 95% CI 1.66–21.41). Conclusions: The findings of our study can contribute to understanding the reasons for the LTFU of patients with TB and can guide policies and facilitate designing measures to allow better adherence and, therefore, greater treatment success.

## 1. Introduction

Tuberculosis (TB) continues to be a public health concern worldwide, representing the second cause of death from a single infectious agent after COVID-19. Worldwide, in 2022, an estimated 10.6 million people developed TB, and 1.3 million died due to the disease. In 2014, the end TB strategy was launched with specific targets for a reduction in TB incidence, death and catastrophic costs [1].

A lost-to-follow-up (LTFU) patient is defined as a TB patient who did not start treatment or whose treatment was interrupted for at least two consecutive months [2]. Patients with TB who are LTFU are at a greater risk of death and drug resistance development [3,4] and can become sources of infection in the community, leading to community outbreaks [5,6,7]. Moreover, LTFU patients increase the cost of TB treatment [8]. Hence, LTFU must be one of the primary concerns in combating TB.

Many factors have been related to LTFU TB patients. Male sex, smokers and patients with alcohol abuse have been consistently found to be most commonly LTFU [9]. Besides individual and behavioural characteristics, social determinants such as a lack of familial support and long distance to the health centres clearly limit adherence to treatment [2]. Similarly, a lack of basic education has been found to be related to treatment abandonment. It has been thought that illiterate people may have more difficulty understanding clinical recommendations and more easily fall into misunderstandings [2]. In addition, it has been hypothesised that most tuberculosis patients with little or no knowledge of infection and disease live in precontemplation, which hinders adherence to treatment and follow-up [4]. In addition, adverse drug events and the length of the treatment facilitate a lack of therapy adherence, especially in those patients with multidrug-resistant TB (MDR-TB) [3].

Angola is one of the 30 countries with the greatest TB and MDR-TB burden worldwide [1]. To date, there are little data about the risk factors for LTFU among TB patients in the country [10]. Therefore, the aim of this study was to identify and analyse the risk factors associated with LTFU of TB treatment in the Sanatorium Hospital in Luanda.

## 2. Methods

This is an observational prospective study, including patients with pulmonary and extrapulmonary TB who started first-line TB treatment in the Sanatorium Hospital in Luanda between 1 August 2018 and 30 September 2019. Patients older than 15 years old who started first-line TB treatment and who signed written informed consent were included. Multidrug-resistant TB (MDR-TB) patients and those who were admitted in a coma were excluded.

Basal data were recorded, and follow-up was performed until the end of treatment or until they were LTFU. Sociodemographic variables included gender, age, place of residence (urban or suburban), distance to the health centre (<10 miles (near) compared to >10 miles (far)), homeownership, transportation fees to the hospital (<600 vs. ≥600 Kwanzas), level of education (basic, medium and university studies) ethnicity (Bakongo, Umbundu, Kimbundu and other), religion (Christianity, Islam, Hindu and no religion), current employment, monthly income (low salary AOA < 80,000 and medium salary AOA > 80,000) and family support during the disease. Moreover, to explore if religion played a role in the outcome of the treatment, participants were asked if they believed that God was a TB healer, if they knew anyone who had stopped treatment due to religious reasons or if they knew someone who was cured by prayer. We also explored the participants’ knowledge about TB, including 7 basic questions about the causative agent, transmission, clinical manifestations, diagnosis, treatment and prevention. Patients were considered to have knowledge about TB if they correctly answered at least 2 questions. Medical history included smoking habits, alcohol consumption, a familiar and personal history of TB, comorbidities, TB symptoms (fever, cough, weight loss, night sweats, asthenia, thoracic pain, dyspnoea, haemoptysis) and the site of TB (pulmonary vs. extrapulmonary). Severe TB was defined as a patient who presented an oxygen saturation below 90% and/or massive haemoptysis.

Regarding the diagnosis of TB, we collected information about AFB sputum smear (negative, scanty (1–9 AFB in 100 fields), 1+ (10–99 AFB in 100 fields), 2+ (1–10 AFB per field), and 3+ (more than 10 AFB per field), Xpert MTB/RIF qualitative and semiquantitative results and chest X-ray results (presence of cavities). Tuberculosis treatment information was also recorded, and adverse events were classified as skin toxicity, arthritis, gastrointestinal symptoms and hepatic toxicity. The outcome of the treatment was defined as per the 2013 WHO recommendations, including categories such as cured and completed treatment (treatment success), LTFU, died, transferred, and failure [11].

### 2.1. Data Analysis

The database was designed in Microsoft Excel, and it was transferred to the SPSS software for Windows (Version 19.0; SPSS Inc, Chicago, IL, USA) for the statistical analysis. Qualitative variables were expressed as absolute numbers and percentages, while quantitative ones were expressed through means and standard deviations (SD) or median and interquartile ranges (IQRs), depending on the distribution. The χ^2^ test or Fisher’s exact test, when appropriate, was used to compare the distribution of categorical variables, and Student’s t-test or Mann–Whitney test for continuous variables. Results were considered statistically significant if the 2-tailed *p*-value was <0.05. For the univariate and multivariate analysis, we excluded those patients who died, were transferred or failed treatment. The variable TB outcome was banded into two groups (cured and LTFU) and considered as the dependent variable. Variables with a *p*-value < 0.20 in the univariate analysis or those considered to be clinically important were included in the multivariate logistic regression analysis. The odds ratios presented in the study have not been adjusted for other variables.

### 2.2. Ethical Considerations

The study was designed, implemented and reported in accordance with the Declaration of Helsinki, Good Clinical Practice guidelines and was approved by the ethics committee of the Ministry of Health of Angola (MINSA) Nº 06/2018.

## 3. Results

### 3.1. Sociodemographic Data and TB Knowledge

A total of 113 patients with TB were included. Sixty-seven (59.3%) were males, and the median (IQR) age was 30 (22–44) years old. Sociodemographic data are presented in Table 1.

The majority of respondents (107, 94.7%) stated that God could heal TB; 16 (14.2%) reported knowing someone who had been cured by prayer, and 8 (7.1%) reported knowing someone who had stopped treatment for religious reasons. Overall, 46 (40.7%) patients knew at least two of the seven questions related to TB.

### 3.2. Clinical Symptoms and Diagnosis

One hundred (88.5%) patients were diagnosed with TB for the first time. The most common TB symptoms were weight loss in 104 (92.0%), fever 103 (91.2%) and cough 103 (91.2%). Ninety-eight (86.7%) cases were diagnosed with pulmonary TB, and 27 (23.9%) presented with severe TB at the moment of diagnosis. Ninety out of the ninety-eight pulmonary TB (91.8%) were able to provide a sputum sample, and 62 (68.8%) had a positive sputum smear. Molecular testing by Xpert MTB/RIF was performed in 22 patients, and all of them had a positive result for TB. A chest X-ray was conducted in 72 (63.7%) patients, and 47 (65.3%) of them presented with lung cavitation. Clinical symptoms and diagnosis data are presented in Table 2.

### 3.3. Treatment and Outcomes

All patients (113, 100%) were treated with the standard first-line four-drug regimen for TB. Twenty-three (20.4%) patients presented at least one adverse event, with the most frequent being gastrointestinal symptoms (14, 60.9%) and skin toxicity (5, 21.7%). Regarding treatment outcomes, 76 (67.3%) patients were cured, 27 (23.9%) were LTFU, 5 (4.4%) died, 4 (3.5%) were transferred and 1 (0.9%) failed treatment.

### 3.4. Variables Associated with LTFU

In the univariate analysis, we observed that men (74.1% vs. 25.9%, *p* = 0.03), those who usually ate less than three meals per day (65.8% vs. 33.3%, *p* = 0.002) and those who presented with severe TB at the time of diagnosis (37.0% vs. 15.8%, *p* = 0.021) were more frequently LTFU. No other sociodemographic, lifestyle or health factors were significantly related to TB outcome. Regarding TB beliefs and knowledge, we observed that people who were cured or believed in God as a TB healer constituted a greater proportion than those who were LTFU (98.7% vs. 88.9%, *p* = 0.024). No significant association was found between having knowledge about TB and treatment outcome (32.9% vs. 22.2%, *p* = 0.299). This information is summarised in Table 3.

In the multivariate analysis, we only observed that those patients with severe TB at the moment of diagnosis and those who usually ate less than three meals per day were significantly associated with LTFU ((OR 9.24, 95% CI 2.18–39.04, *p* = 0.006) and (OR 5.96, 95% CI 1.66–21.41, *p* = 0.006), respectively) (See Table 4).

## 4. Discussion

In this prospective observational study, evaluating factors associated with the LTFU of TB treatment in Luanda, we described a high proportion of patients who were LTFU (23.9%). We noted that those patients with severe TB at presentation and those who ate less than three times a day were more likely to be LTFU. In our study, treatment success was lower than expected compared to other experiences in the sub-Saharan African region (82% to 92%) [12,13,14]. Our LTFU figures are higher than those reported by the WHO Global Report 2023 for people treated with first-line anti-TB drugs [1]. Undoubtedly, this low treatment success is related to the high percentage of LTFU and dropout observed in our study.

When we analysed the risk factors related to LTFU, we found that being male was significantly associated with a risk of discontinuing treatment, although statistical significance was lost in the multivariate analysis. Despite this, being male has consistently been found as a risk factor for LTFU. As there is no biological reason that can be linked to the higher dropout rate among men, the most common explanations have been related to the social and cultural sphere. As an example, Santos et al. observed that, in Angolan society, as well as other cultures in the region, there is a higher social pressure on men to be the economic driver to support the family. This fact could justify the fact that men could be less adherent to medical follow-up and treatment [10]. Another explanation is that men usually consume more alcohol or other illicit drugs than women, which are habits widely described as risk factors for therapeutic non-compliance [15].

As Angola is a country with a strong Christian faith, we wanted to investigate if religion played a role in TB treatment and its outcome. In this regard, we observed that believing in God as a TB healer was significantly associated with treatment success (*p* = 0.024), although this association was lost in the multivariate analysis. It has already been described how many individuals in Angola continue to believe that Gods and other supernatural entities have a role in their physical and mental health [16]. According to Perdigao et al., the majority of Christians agreed with the idea that God can cure TB [17]. In nations where religious views are highly valued, the cooperation of community and religious leaders can contribute to TB control. This permits a more effective and culturally appropriate outreach to at-risk populations [16].

On the other hand, we observed that severe TB at the moment of diagnosis was associated with LTFU in the multivariate analysis. One of the most plausible explanations for this finding is that when patients are seriously ill or do not improve, their relatives or the patients themselves prefer to administer traditional medicine and then become LTFU [18]. In addition, there may be family conflicts after the death of a patient in the hospital, and families may prefer to have the patient die at home [19]. Moreover, patients with more severe diseases tend to be admitted for longer periods of time, thus consuming more economic resources, making it easier to incur catastrophic costs and, therefore, making it more difficult for patients to continue outpatient follow-up once they are discharged [20].

We noted that those patients who could not guarantee three meals per day were more often LTFU. We believe that, in this case, the number of meals per day is a proxy for poverty. There is a reciprocal relationship between poverty and TB. Poverty may be related to poor sanitary conditions that increase susceptibility to TB, and the disease limits work and livelihood opportunities, thus forming a vicious circle that tends to worsen the severity and negative impact of the disease. TB is related to unfavourable socioeconomic conditions, which usually worsen when a member of the household is ill. Catastrophic costs associated with TB are present in more than 40% of families affected by the disease and frequently affect the most vulnerable populations [21]. Regardless of medicines for TB being free in most settings, including Angola, there are some expenses that the patients still incur, such as consultation fees, laboratory tests, and other indirect costs, such as transportation and family accommodation. Moreover, in Angola, main meals during hospital admission are not paid for by the health system and have to be afforded by patients. In the same way, in Angola, most patients do not have health insurance and have no income while they are sick and unable to work. This condition indicates that patients have to distinguish and prioritise between treatment and food, respectively [22]. Unfortunately, we did not find any other relation between LTFU and other surrogate markers of poverty (e.g., salary).

There are several limitations to our study. Firstly, the number of patients included during the study period was lower than expected, which may have limited obtaining more robust evidence, especially regarding variables related to LTFU. This was due to changes in the management of the Luanda Sanatorium Hospital and the difficulty of being in the country to recruit participants. Secondly, some of the patients included in the study were diagnosed with TB without microbiological confirmation, so it is possible that some did not actually have TB but another respiratory infection that could have negatively influenced the outcome of the treatment. Finally, we were unable to track whether LTFU patients were treated in another health centre due to the lack of computerisation and communication between health units.

In conclusion, our findings show that a high proportion of patients are LTFU during TB treatment in Luanda. Moreover, we describe how disease severity and food insecurity are significantly related to higher LTFU rates. These findings reinforce the fact that the fight against poverty is clearly necessary to end TB.

## Figures and Tables

**Table 1:** Sociodemographic characteristics.

	*N* = 113
Sex (male)	67 (59.3%)
Age (years)	30 (22–44)
**Location**	
Urban	23 (20.4%)
Suburban	90 (79.6%)
House ownership	27 (23.9%)
**Transportation fee**	
AOA < 600	94 (83.2%)
AOA ≥ 600	19 (16.8%)
**Education**	
Basic incomplete	39 (34.5%)
Basic complete	21 (18.6%)
Medium incomplete	27 (23.9%)
Medium complete	19 (16.8%)
University	7 (6.2%)
**Ethnicity**	
Bakongo	23 (20.4%)
Umbundu	33 (29.2%)
Kimbundu	51 (45.1%)
Other	6 (5.3%)
**Religion**	
Christianity	107 (94.7%)
Islam	1 (0.9%)
Hindu	1 (0.9%)
No religion	4 (3.5%)
Employment	46 (40.7%)
**Salary** (*n* = 46)	
Low (AOA < 80,000)	40 (86.9%)
Medium (AOA ≥ 80,000)	6 (13%)
Smoking	23 (20.4%)
Alcohol abuse	39 (34.5%)
**Comorbidities**	
Asthma	7 (6.2%)
Diabetes	13 (11.5%)
HIV	22 (19.5%)
Chronic heart failure	8 (7.1%)
Hypertension	34 (30.1%)
Malaria infection	39 (34.5%)
**Number of meals per day**	
Less than three meals	50 (44.2%)
Three meals	63 (55.8%)

Values are expressed as the number and percentage or median [IQR] unless otherwise specified. HIV: Human Immunodeficiency Virus.

**Table 2:** Clinical presentation, diagnosis, follow-up and outcomes.

	*N* = 113
History of TB in the family	26 (23%)
**TB patient**	
New case	100 (88.5%)
Previously treated	13 (11.5%)
**Symptoms and signs**	
Fever	103 (91.2%)
Cough	103 (91.2%)
Weight loss	104 (92%)
Night sweats	78 (69%)
Asthenia	73 (64.6%)
Thoracic pain	49 (43.4%)
Dyspnea	37 (32.7%)
Hemoptysis	17 (15%)
Adenopathies	7 (6.2%)
Ascites	2 (1.8%)
Spinal column deformation	9 (8%)
**AFB sputum smear**	90 (79.6%)
Positive	62 (68.9%)
**Xpert MTB/RIF**	22 (19.5%)
Positive	22 (100%)
**Chest X-ray**	72 (63.7%)
Cavitation	47 (65.3%)
**Severe TB**	27 (23.9%)
Type of TB	
Pulmonary	98 (86.7%)
Extrapulmonary	13 (11.5%)
Pulmonary and extrapulmonary	2 (1.8%)
**Adverse events** (No. of patients presenting at least one)	23 (20.4%)
Skin toxicity	5 (21.7%)
Arthritis	1 (4.3%)
Gastrointestinal symptoms	14 (60.9%)
Liver toxicity	3 (13%)
**Treatment outcome**	
Cured	76 (67.3%)
Lost to follow-up	27 (23.9%)
Died	5 (4.4%)
Transferred	4 (3.5%)
Failure	1 (0.9%)

Values are expressed as the number and percentage unless otherwise specified. TB: tuberculosis.

**Table 3:** Univariate analysis of risk factors and LTFU.

***N*** ** = 103**	**Cured (76, 73.8%)**	**LTFU (27, 26.2%)**	***p*** **-Value**
Sex (male)	38 (50%)	20 (74.1%)	0.03
Age (years)	28 (21–41)	35 (25–46)	0.22
**Area of residence**			
Urban	14 (18.4%)	6 (22.2%)	0.22
Suburban	62 (81.6%)	21 (77.8%)	0.22
House ownership	18 (23.7%)	6 (22.2%)	0.87
Family support	64 (84.2%)	19 (70.4%)	0.87
**Transportation fee**			
AOA ≤ 600	64 (84.2%)	22 (81.5%)	0.74
AOA > 600	12 (15.8%)	5 (18.5%)	
**Education**			
Basic incomplete	26 (34.2%)	10 (37.1%)	
Basic complete	15 (19.7%)	6 (22.2%)	
Medium incomplete	17 (22.4%)	6 (22.2%)	0.95
Medium complete	12 (15.8%)	4 (14.8%)	
University	6 (7.9%)	1 (3.7%)	
**Ethnicity**			
Bakongo	17 (22.4%)	5 (18.5%)	
Umbundu	23 (30.3%)	8 (29.6%)	0.93
Kimbundu	32 (42.1%)	13 (48.1%)	
Other	4 (5.3%)	1 (3.7%)	
**Religion**			
Christianity	75 (98.7%)	24 (88.9%)	
Islam	0	1 (3.7%)	0.09
Hindu	0	1 (3.7%)	
No religion	1 (1.3%)	1 (3.7%)	
Employment	29 (38.2%)	10 (37%)	0.91
**Salary**			
No salary	47 (61.8%)	17 (63%)	
Low salary	24 (31.6%)	9 (33.3%)	0.85
Medium salary	5 (6.6%)	1 (3.7%)	
Smoking	12 (15.8%)	7 (25.9%)	0.24
Alcohol abuse	21 (27.6%)	11 (40.7%)	0.21
**Comorbidities**			
Asthma	4 (5.3%)	2 (7.4%)	0.68
Diabetes	6 (7.9%)	3 (11.1%)	0.61
HIV	15 (19.7%)	7 (25.9%)	0.5
Chronic heart failure	3 (3.9%)	2 (7.4%)	0.47
Hypertension	20 (26.3%)	9 (33.3%)	0.48
Malaria	26 (34.2%)	8 (29.6%)	0.66
**Number of meals per day**			
Less than three	26 (34.2%)	18 (66.6%)	0.002
Three meals	50 (65.8%)	9 (33.3%)	
**Religious beliefs**			
God can heal TB	75 (98.7%)	24 (88.9%)	0.02
Someone stops treatment for religion	5 (6.6%)	2 (7.4%)	0.08
Cured by a prayer	13 (17.1%)	3 (11.1%)	0.46
TB knowledge	25 (32.9%)	6 (22.2%)	0.29
Familiar TB history	18 (23.7%)	8 (29.6%)	0.54
**Symptoms and signs**			
Fever	69 (90.8%)	24 (88.9%)	0.77
Cough	67 (88.2%)	26 (96.3%)	0.22
Weight loss	7 (9.2%)	2 (7.4%)	0.77
Night sweats	48 (63.2%)	21 (27.6%)	0.16
Asthenia	46 (60.5%)	19 (70.4%)	0.36
Thoracic pain	36 (47.4%)	7 (25.9%)	0.05
Dyspnea	27 (35.5%)	6 (22.2%)	0.2
Hemoptysis	12 (15.8%)	2 (7.4%)	0.27
Adenopathies	5 (6.6%)	1 (3.7%)	0.58
Ascites	2 (2.6%)	0	0.39
Column deformity	7 (9.2%)	2 (7.4%)	0.77
**AFB sputum smear**			
Positive	41 (53.9%)	15 (55.6%)	
Negative	21 (27.6%)	6 (22.2%)	0.82
Not performed	14 (18.4%)	6 (22.2%)	
**AFB sputum smear quantification**			
0	35 (46.1%)	12 (44.4%)	
1+	12 (15.9%)	6 (7.9%)	0.89
2+	20 (26.3%)	6 (7.9%)	
3+	9 (11.8%)	3 (11.1%)	
**Chest X-ray**			
Cavitation	33 (50%)	11 (16.7%)	0.81
Severe TB	12 (15.8%)	10 (37%)	0.02
**Type of TB**			
Pulmonary	66 (86.8%)	25 (92.6%)	
Extrapulmonary	8 (10.5%)	2 (7.4%)	0.98
Pulmonary and extrapulmonary	2 (2.6%)	0	
**Type of TB patient**			
New case	69 (90.8%)	22 (81.5%)	0.19
Previously treated	7 (9.2%)	5 (18.5%)	
Adverse events	15 (19.7%)	6 (7.9%)	0.78
**Type of adverse events**			
Skin toxicity	4 (5.3%)	1 (1.3%)	
Arthritis	1 (1.3%)	0	0.12
Gastrointestinal toxicity	10 (13.2%)	3 (3.9%)	
Liver toxicity	0	2 (2.6%)	

Values are expressed as the number and percentage or median [IQR] unless otherwise specified. HIV: Human Immunodeficiency Virus, AFB: Acid Fast Bacilli, and TB: tuberculosis.

**Table 4:** Multivariate analysis of risk factors and LTFU.

	Cured (76, 73.8%)	LTFU (27, 26.2%)	OR	95% CI	*p*-Value
Gender (male)	38 (50%)	20 (74.1%)	3.32	0.9–12.1	0.091
Eat less than 3 times a day	26 (34.2%)	18 (66.6%)	5.96	1.66–21.41	0.006
Belief in God as a healer of TB	75 (98.7%)	24 (88.9%)	0.24	0.02–70.01	0.251
Previously treated patients	7 (9.2%)	5 (18.5%)	0.54	0.09–3.12	0.493
Severe TB	12 (15.8%)	10 (37%)	9.24	2.18–39.04	0.002
Knowledge about TB	25 (32.9%)	6 (22.2%)	0.25	0.04–1.34	0.107

## Data Availability

The data presented in this study are available on request from the corresponding author.
